# An Experimental and Theoretical Study on Essential Oil of *Aethionema sancakense*: Characterization, Molecular Properties and RDG Analysis

**DOI:** 10.3390/molecules27186129

**Published:** 2022-09-19

**Authors:** Azize Demirpolat, Feride Akman, Aleksandr S. Kazachenko

**Affiliations:** 1 Vocational School of Food, Agriculture and Livestock, University of Bingöl, Bingöl 12000, Turkey; 2 Krasnoyarsk Scientific Center, Department of Chemistry of Natural Organic Raw Materials, Institute of Chemistry and Chemical Technology, Siberian Branch, Russian Academy of Sciences, Akademgorodok, 50, bld. 24, Krasnoyarsk 660036, Russia; 3 Department of Non-Ferrous Metals and Materials Science, Siberian Federal University, pr. Svobodny 79, Krasnoyarsk 660041, Russia; 4 Department of Biological Chemistry with Elements of Pharmaceutical, Medical and Toxicological Chemistry, Krasnoyarsk State Medical University of the Ministry of Healthcare of the Russian Federation, st. Partizan Zheleznyak, bld. 1, Krasnoyarsk 660022, Russia

**Keywords:** *Aethionema sancakense*, linoleic acid, α-humulene, camphene, heptanal, DFT

## Abstract

This study aims to experimentally and theoretically examine the plant *Aethionema sancakense*, which was determined as a new species and whose essential oil and fatty acid compositions were characterized by GC/GC-MS technique. Linoleic acid (23.1%), α-humulene (19.8%), camphene (13.9%), and heptanal (9.7%) were found to be the major essential oil components of *A. sancakense* aerial part structures. The quantum chemical calculations of these four molecules that are very important to this plant were performed using the density functional method (DFT)/B3LYP with the 6-31 G (d, p) basis set in the ground state for the gas phase. The molecular structures, HOMO-LUMO energies, electronic properties, Fukui functions, and molecular electrostatic potential (MEP) surfaces of the major constituents of *Aethionema sancakense* essential oil were calculated and interpreted. Finally, the RDG-NCI analysis of these molecules was performed to determine the non-covalent interactions present within the molecules.

## 1. Introduction

The Brassicaceae family, to which the *Aethionema sancakense* Yıld. and Kılıç species belongs, is a broad family consisting of 345 genera and 4020 species, spread in all continents in the world except Antarctica [1]. It is estimated that *Aethionema* currently includes about 53 recently described species in Turkey [2]. Yıldırımlı and Kılıç proposed *Aethionema sancakense* as a new species belonging to Brassicaceae [3]. Members of the Brassicaceae family are valuable species in terms of fatty acids. Studies on the fatty acid profile of *Aethionema* species have reported that *Aethionema grandiflora* contains 63.60%, 10.9%, and 11.80% of linolenic, oleic, and linoleic acids, respectively [4]. Another species from the same family, *A. saxatile* from the genus *Alyssoides*, contains 56.4%, 14.9%, and 8.7% of these fatty acids, respectively, and *Arabidopsis thaliana* from the genus *Arabidopsis* contains 55.5%, 13.5%, and 16.7% [4]. These fatty acids differ between species in the Brassicaceae family. The phytochemistry of *Aethionema* species has been little studied. After studies that have determined the presence of alkaloids in *Aethionema* species [5], the presence of kalistegins was also identified in these species [6]. Twelve different volatile molecules were detected in *A. diastrophis* extracts together with chlorogenic acid, protocatechoic aldehyde, and benzoic acid. For another study conducted on *A. armenum* extracts, flavonoid glycosides such as quercitrin and afzelin have been isolated and the chemotaxonomic importance of these compounds has been revealed [7]. It has been proven by studies that *Aethionema* species produce high levels of glucosinolate [8,9,10,11].

In folk medicine, species belonging to the genus *Aethionema* are used against typhoid, bacterial infections, and meningitis [12]. Another study investigating the phenolic content and antioxidant activity of 30 plants selected using the DPPH scavenging method indicated that the highest phenolic content was determined in *Aethionema devmonii* [13]. Aliyazicioglu et al. [14] investigated the essential oil compounds of *A. diastrophis* extracts, observing that this species exhibits a high antioxidant and antimicrobial capacity and suggesting that *A. diastrophis* may be suitable for use as a raw material in drug, food, and perfume industries. Secondary metabolites obtained from plants are important in terms of phenolics and flavonoids as well as having natural antioxidant properties [15] (Denniston et al., 2007). These compounds, including natural and synthetic antioxidants, are substances that, thanks to their radical scavenging properties, can interact with free radicals and protect cells against oxidant damage and offer beneficial effects on health [16]. Accordingly, there has recently been an increasing interest in the production of certain herbal therapeutics with biological functions for the treatment of various diseases [17,18]. Density functional theory (DFT) method, which has been very preferred in recent years, is frequently used in molecules because it gives accurate results close to experimental values [19,20,21,22]. DFT is often used as a powerful tool for accurate predictions of molecular geometries required for reliable studies of molecular properties in a short time [23].

Moreover, density-functional theory (DFT) is a computational quantum mechanical modeling method for describing the ground state properties of atoms, molecules, and solids based on electron densities. Using this theory, the properties of a multi-electron system can be determined using functionals such as the B3LYP method, the most famous hybrid density functional theory model, which includes Hartree–Fock variation, gradient variation correction, gradient correlation correction, local variation, and local correlation [24]. Known as the most popular DFT method, B3LYP represents the most famous global hybrid generalized gradient approach (hybrid-GGA) and is widely used in almost every field of chemistry and has dominated the DFT market for nearly 20 years [24].

The study of the main components of essential oils by theoretical methods (in particular, DFT) is actively spread. Density-functional theory calculations for monomeric substances from essential oils allow us to draw conclusions about their molecular properties, charge distribution, chemical activity, and other important characteristics. Thus, in the work [25], the authors studied the seasonal changes in the components of essential oils *Piper cernuum* Vell and *Piper rivinoides* Kunth. In the article [26], the authors studied the main components of essential oils from *Lippia sidoides*.

Therefore, in this study, we aimed to understand the predominant molecules in the essential oil composition of the plant more closely and to give preliminary information about the molecules with the B3LYP method. Although there have been some theoretical studies on these molecules, we have not encountered any studies in line with our different perspective. Therefore, this inadequacy of information in the literature encouraged us to undertake a theoretical study on the major constituents of *Aethionema sancakense* essential oil. Accordingly, in this study, *A. sancakense*, which was defined as a new species, was characterized by both molecular and electronic properties, Fukui functions, and molecular electrostatic potential (MEP) surfaces; and the RDG analysis of linoleic acid, α-humulene, camphene, and heptanal, which are the major constituents of its essential oil, were determined theoretically.

## 2. Materials and Methods

### 2.1. Collection of the Plant Material

The plant material used in the study was collected from Bingöl province. It was collected from its natural habitat in its intense flowering period in May–June, which is the flowering period of the plant, and dried in the shade. The specimens of the plant are preserved in the Bingol University. 

Type. Turkey. BİNGÖL: Center, 7 km towards Aşağıköy, oak forest clearing, slope, 1450–1550 m. 2020. Latitude 38.856393, Longitude 40.373287.

### 2.2. Isolation of the Essential Oil 

Aboveground parts of *A. sancakense* (100 g) were hydrodistilled for 3 h using a Clevenger type apparatus. For fatty acid analysis, the first 5 g of each plant sample was homogenized in 10 mL of liquid. The sample in hexane/isopropanol was centrifuged at 10,000 rpm for 30 s and 10 min at 5000 rpm [25]. The upper part was taken and placed into test tubes by filtering. Fatty acids need to be derivatized to be able to analyzed by GC. For this purpose, they are usually derivatized with methyl esters. The method proposed by Christie for derivatization was used in the study in Bingol University Central Laboratory Application and Research Center [26]. Five milliliters of 2% methanolic sulfuric acid was added to the sample and vortexed. Then, the sample was kept at 50 °C for 15 h for methylation to occur. After this step, the tubes were cooled to room temperature and 5 mL of 5% NaCl was added. Fatty acid methyl esters formed in test tubes were extracted with 5 mL of hexane; the hexane phase was removed from the top using a pasta pipette; then, the residue was treated with 5 mL of 2% KHCO_3_. After waiting 1 h, the resulting mixture was evaporated from its solvent under nitrogen flow at 45 °C. The fatty acids found in the tubes were dissolved in 1 mL of hexane and made ready for analysis.

### 2.3. Gas Chromatography-Mass Spectrometry (GC-MS) and FID Analysis 

The essential oil was analyzed using the HP 6890 GC equipped with an FID detector. Column and analysis conditions were the same as in GC-MS. The essential oil compounds were identified using the Wiley and Nist mass spectral library, and the identified compounds of the essential oil are listed in Table 1. An Agilant 7890A/5970 C GC-MS instrument with an SGE Analytical BPx 90 100 m × 0.25 mm × 0.25 µm column was used for fatty acid analysis. The temperature program was adjusted to gradually heat from 120 °C to 250 °C in a total of 45 min. The samples were washed five times in hexane. The injection volume of 1 µL, the split ratio of 10:1, solvent delay time of 12 min, the carrier gas flow rates of 35 mL/min for He, 350 mL/min for H_2_, and 20.227 mL/min for N_2_ were automatically determined by the program. The fatty acid compounds identified in the studied taxa are presented in Table 2.

## 3. Result and Discussion

### 3.1. Analysis of Essential Oil

In this research, essential oil and fatty acid compositions of *Aethionema sancakense* Yıld. and Kılıç were analyzed by GC/GC-MS. Linoleic acid (23.1%), α-humulene (19.8%), camphene (13.9%), and heptanal (9.7%) were found to be the major essential oil components of *A. sancakense* aerial part structures. The average major fatty acid compositions of *A. sancakense* oil were linoleic acid (55.38%), palmitic (14.70%), linoleic acid (9.26%), and steraic (2.76%), whereas other fatty acids were found in small proportions. The study that was carried out to determine the major volatile components of *A. diastrophis* revealed that the most abundant monoterpene hydrocarbons were camphene (21.7%) and α-humulene (18.3%) [14]. In our study, α-humulene and camphene were found to be 19.8% and 13.9%. The results were in agreement with each other when compared in terms of the major components of the essential oils. The study to determine the major volatile components of *A. diastrophis* revealed that the most abundant monoterpene hydrocarbons are camphene (21.7%) and α-humulene (18.3%) [14]. 

**Figure 1:**
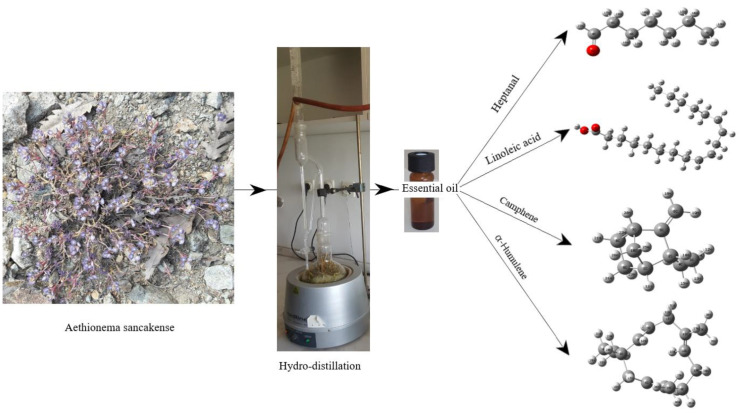
Analysis scheme for essential oil contents of the *Aethionema sancakense* plant.

In our study, α-humulene and camphene were found to be 19.8% and 13.9%. When the essential oils were compared in terms of the main components, it was seen that the results were compatible with each other. Looking at the studies on *Aethionema* in the literature, the fatty acid profile of *A. pulchellum* seeds has been investigated. The FAS percentages were determined as follows: 16:0–7:0%; 16:1–0.5%; 18:0–1.3%; 18:2–12.9%; and 18:3–67.2% [27]. Linoleic acid was found to be C18:2 (55.38%) in our study. In another study examining the sterol composition, cholesterol (8.8), campestreol (6.5), stigmastreol (4.5), and β-cytostreol (85.2) were determined [28]. The composition of sterols in *Aethionema schistosum* was found to be 4.0% cholesterol, 6.0% campesterol, 1% stigmasterol, and 89.0% β- cytostreol. As a result, *A. sancakense* is a newly identified species whose chemical properties have not been studied before. The species was found valuable in terms of essential oil and fatty acid composition and the analysis scheme for the essential oil content of it is given in Figure 1. Although there are a limited number of studies on the genus *Aethionema*, some qualitative and quantitative differences were observed in *A. sancakense* when compared with the results of other studies. There are differences between species in terms of chemical properties due to genetic characteristics of the examined plant parts, environmental factors, and analytical methods used.

### 3.2. Computational Details

**Figure 2:**
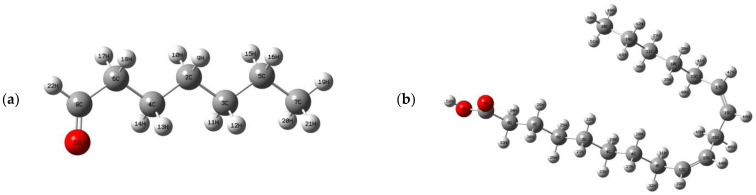

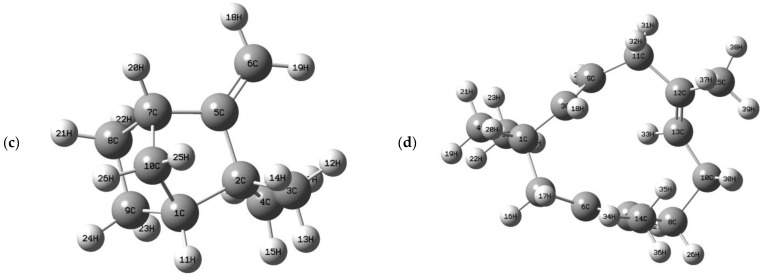
Optimized molecular structures of heptanal (**a**), linoleic acid (**b**), camphene (**c**), and α-humulene (**d**) molecules in essential oil.

For the theoretical study, linoleic acid, α-humulene, camphene, and heptanal molecules, which are the major constituents of *Aethionema sancakense* essential oil, were selected and the theoretical study was carried out. All DFT calculations were made using the Windows version of the Gaussian 09W [29] and GausView 0.5 [30] molecular imaging program. The applied methods are the B3LYP hybrid functional (Becke’s three-parameter hybrid functional (B3)) [31] combined with the Lee–Yang–Parr functions (LYP) [32] and 6-31 G (d, p) basis set. First, the geometric optimization and frequency calculations of the major constituents of the studied plant essential oils were performed using the density-functional theory (DFT) method. The obtained vibration frequencies are all positive, indicating that all fixed points on the potential energy surface are found as true minimums. The optimized molecular structures of heptanal (a), linoleic acid (b), camphene (c), and α-humulene (d) molecules are shown in Figure 2. Secondly, the molecular electrostatic potential (MEP) and frontier moleculer orbital (FMO) analyses were performed to determine the electronic properties and sites of nucleophilic and electrophilic attacks of studied molecules. Finally, the Fukui functions and RDG analysis of these molecules were determined theoretically.

### 3.3. HOMO-LUMO Analysis

**Figure 3:**
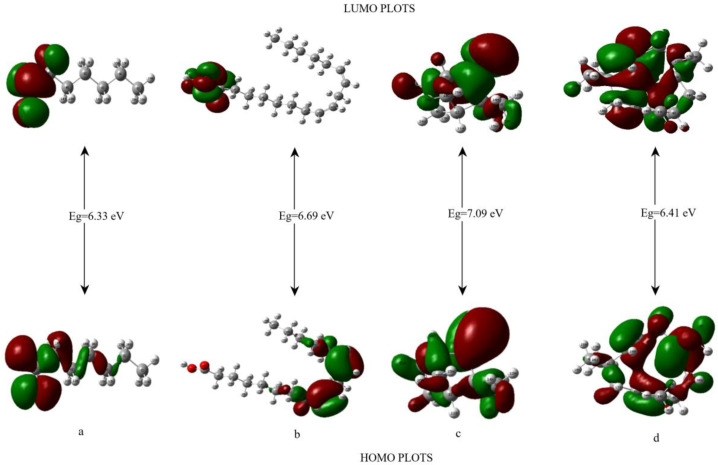
Three-dimensional HOMO LUMO orbital diagram of the studied molecules (**a**–**d**).

Frontier molecular orbitals (FMOs), known as the lowest unoccupied molecular orbital (LUMO) and highest occupied molecular orbital (HOMO), are very important for quantum chemistry, and the energy difference between them plays an important role in determining chemical reactivity, biological activity, kinetic stability, and electronic and optical properties of molecules [33,34]. Three-dimensional plots of the HOMO and LUMO orbitals for the essential oil contents are indicated in Figure 3. It can be seen that from Figure 3, the molecular orbitals for molecules c and d are mostly located on the whole molecule. HOMO and LUMO energies (E_HOMO_ and E_LUMO_), energy gap (Eg), electronegativity (χ), electron affinity (EA), softness (ζ), chemical potential (μ), ionization potential (IP), hardness (η), global electrophilicity index (ω), maximum charge transfer index (∆N_max_), and nucleophilicity index (N) for the heptanal, linoleic acid, camphene, and α-humulene molecules have been computed at the B3LYP/6-31 G (d, p) basis set in the gas phase and water. The data are given in Table 3. These quantum chemical descriptors, determined with the help of HOMO and LUMO energies, can be defined as: [35,36,37]

(1) $$\mathrm{IP}=-E_{\mathrm{HOMO}}$$

(2) $$\mathrm{EA}=-E_{\mathrm{LUMO}}$$

(3) Eg= IP−EA

(4) χ=12EA+IP

(5) η=12IP−EA

(6) $$\zeta =\frac{2}{\left(\mathrm{IP}-\mathrm{EA}\right)}$$

(7) μ=−12EA+IP

(8) $$\omega =\frac{\mu^{2}}{2\eta }$$

(9) $$∆N_{\max}=-\frac{\mu }{\eta }$$

(10) $$N=\frac{1}{\omega }$$

The band gap energy value of the heptanal (a), linoleic acid (b), camphene (c), and α-humulene (d) molecules were calculated as 6.3332, 6.6905, 7.0992, and 6.4197 eV, respectively.

The smaller the energy gap, the more reactive and less stable that molecule [33], and according to these values, we can say that the most reactive molecule is the 1st molecule (a), and the order is as follows: a > d > b > c in the gas phase and water. A large energy gap or large hardness value indicates a hard molecule, a small energy gap or large softness value indicates a soft molecule, and it is seen that the energy gap, hardness, and softness values are compatible with each other. In this case, we can say that the softest molecule is molecule a and the hardest is molecule c. According to the electrophilic index (ω) scale for organic molecules recommended by Domingo et al. [38], if the electrophilic index value is greater than 1.5 eV, it is a strong electrophile, if less than 0.8 eV, it is a weak electrophile, and if it is greater than 0.8 and less than 1.5 eV, it is a medium electrophile. The value of the electrophilic index and the maximum charge transfer index give us information about the binding ability of that molecule with biomolecules [39], and according to Table 3, we can say that molecule a has the highest binding capacity to biomolecules and is the best electrophile compared to other molecules (ω = 2.1362 and 2.1887 eV, ∆N_max_ = 1.1616 and 1.1694 eV in the gas phase and water, respectively).

### 3.4. MEP Analysis

**Figure 4:**
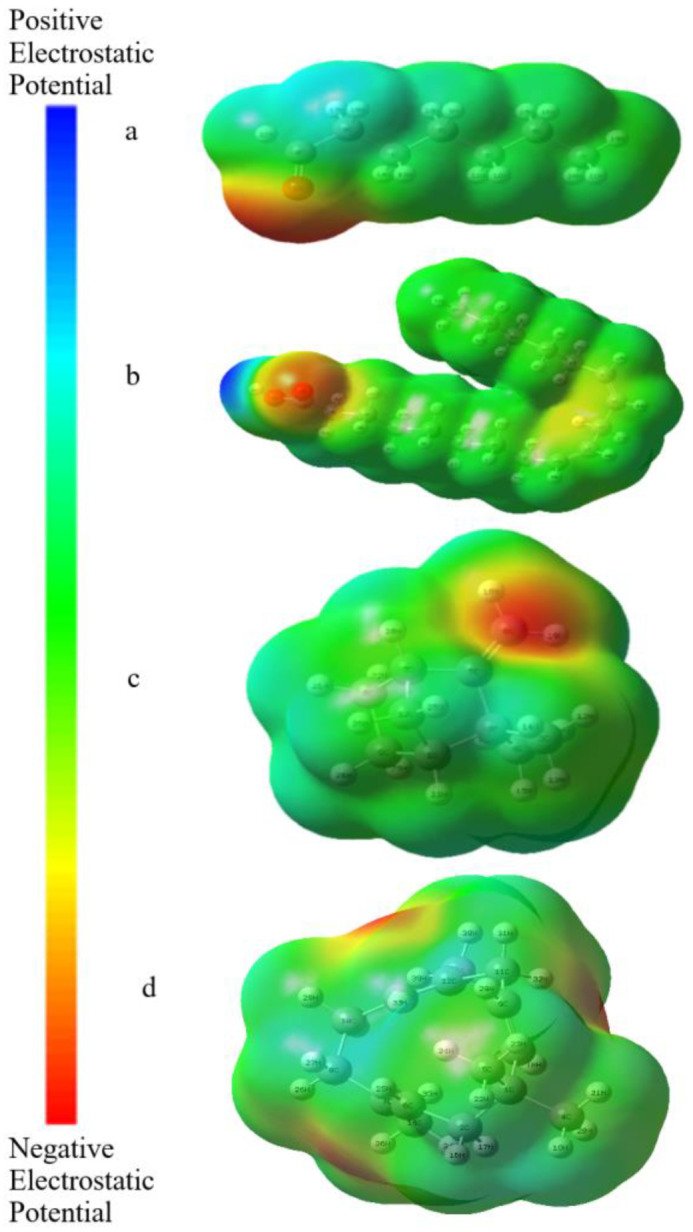
Three-dimensional MEP surface maps of the studied molecules (**a**–**d**).

In the presented study, to understand the change of electron density and chemical reactivity for the electrophilic and nucleophilic attack of the heptanal, linoleic acid, camphene, and α-humulene molecules, molecular electrostatic potential (MEP) surfaces plotted using B3LYP/6-31 G (d, p) method and three-dimensional surface maps are shown in Figure 4. The color codes of MEP maps for the heptanal, linoleic acid, camphene, and α-humulene molecules range from −0.0484 to 0.0484 a.u., range from −0.0553 to 0.0553 a.u., range from −0.0215 to 0.0215 a.u., and range from −0.0216 to 0.0216 a.u., respectively.

Molecular electrostatic potential (MEP) can be thought of as a tool for investigating the charge distribution of atoms on the surface of the molecule and is related to electronic density. Its maps provide an insight into the biological recognition processes, hydrogen bond interactions, and the reactivity of a wide variety of chemical systems in nucleophilic and electrophilic reactions [40]. MEP maps show different colors due to various electrostatic potentials on the surface. The decreasing order of electrostatic potential is blue > green > yellow > orange > red. In the MEP maps, the red-colored regions show the most negative electrostatic potential, the green-colored regions show the zero potential, and the blue-colored regions show the most positive electrostatic potential. The red- and yellow-colored regions (negative electrostatic potential) on the MEP surface are associated with electrophilic reactivity, while the blue-colored regions (positive electrostatic potential) of MEP are associated with nucleophilic reactivity. Looking at Figure 4a, the hydrogen atoms (17H, 18H, and 22H) attached to the carbonyl group have a positive electron density and are shown in blue, while the oxygen atom (1O) in the carbonyl group has a negative electron density and is shown in red. In Figure 4b, the hydrogen atom (52H) attached to the oxygen atom has the most positive electron density and is shown in blue, while the oxygen atom in the carbonyl group (2O) has the most negative electron density and is shown in red. In Figure 4c,d, the most negative electron density is on the vinyl groups and is shown in red.

### 3.5. RDG Analysis of Some Major Constituents of Essential Oil Contents

RDG analysis was used as a useful method to study non-covalent interactions for the heptanal, linoleic acid, camphene, and α-humulene molecules. The reduced density gradient (RDG) analysis was performed by using the non-covalent interactions (NCI) theory and using the Multiwfn (Multifunctional Wavefunction Analyzer) [41] and VMD (Visual Molecular Dynamics) programs [42]. The non-covalent interaction index (NCI) is based on the reduced density gradient (RDG) and is used both to evaluate the nature of weak interactions and to characterize intramolecular or intermolecular interactions. The reduced density gradient (RDG) is a fundamental dimensionless quantity consisting of density and the first derivative and is expressed by the following formula: [43]

RDGr=123π21/3∇ρrρr43

The electron density value of the RDG versus sign (λ_2_) ρ peaks gives us the RDG scatter plot (sign(λ2)ρ is the second Eigen value of the electron density). This graph provides information about the strength and nature of the interaction in the molecule. If [44]

(1) the sign (λ_2_) ρ > 0 : a repulsive interaction (non-bonded)
(2) the sign (λ_2_) ρ < 0 : an attractive interaction (bonded)
(3) the sign (λ_2_) ρ ≈ 0 : a Van der Waals weak interaction

**Figure 5:**
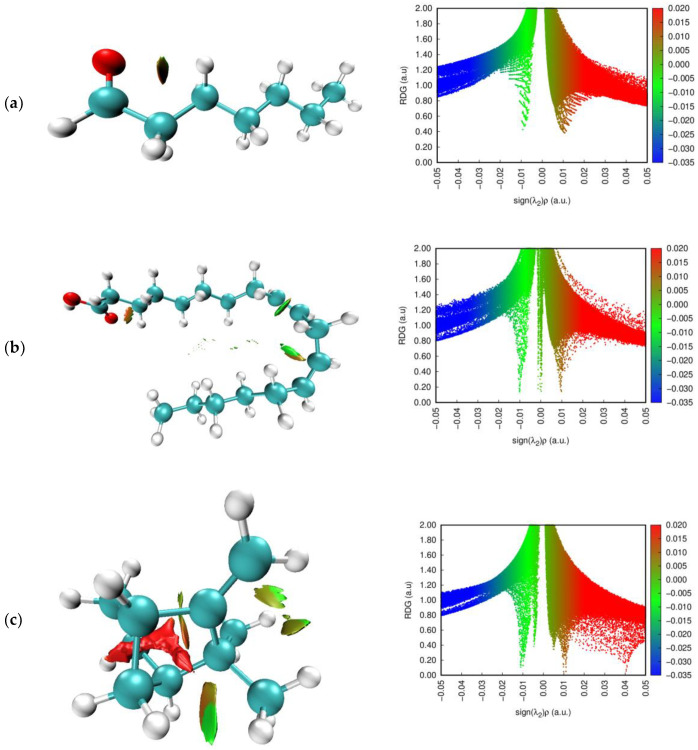

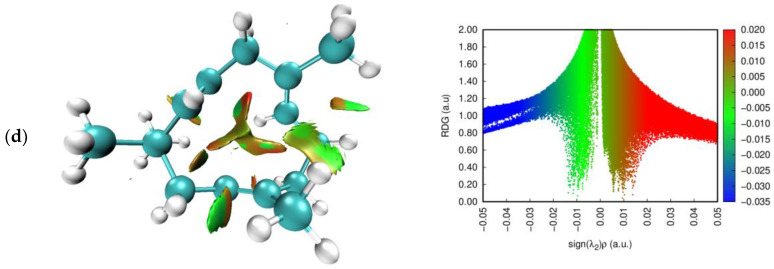
NCI (left) and RDG (right) plots of the studied molecules (**a**–**d**).

The gradient scatter graphs (right) and non-covalent interactions (left) for heptanal, linoleic acid, camphene, and α-humulene molecules are shown in Figure 5. According to Figure 5 (RDG-NCI plots), blue, green, and red colors indicate hydrogen bond interaction, van der Waals interactions, and destabilizing steric interactions, respectively. The results show that while van der Waals interactions are dominant in all molecules, the steric effect is quite dominant in molecule c.

### 3.6. Fukui Functions of Some Major Constituents of Essential Oil Contents

The condensed Fukui functions are determined to characterize the regioselectivity of an atom ‘r’ in a molecule. For this purpose, Fukui functions (f^+^(r), f^−^(r), f^0^(r), Δf(r)) were calculated by using the Multiwfn (Multifunctional Wavefunction Analyzer) [41]. The Fukui functions described by Kolandaivel et al. can be calculated as in the following equations [45]

f^−^(r) = q_(N)_ (r) − q_(N−1)_ (r) (for electrophilic attack)

f^+^(r) = q_(N+1)_ (r) − q_(N)_ (r) (for nucleophilic attack)

f^0^(r) = 1/2 [q_(N+1)_ (r) − q_(N−1)_ (r)] (for radical attack)

where q_(N)_ (r) is the atomic charge on site ‘r’ in a neutral system; q_(N__−1)_ (r) is the atomic charge on site ‘r’ in a cationic system; and q_(N+1)_ (r) is the atomic charge on site ‘r’ in an anionic system. Additionally, the dual identifier Δf(r) is the difference between nucleophilic and electrophilic attacks at a specific site and can be calculated according to the following equation [46].

Δf(r) = f^+^(r) − f^−^(r)

If this value is negative, the specific site could be an electrophilic attack; if it is positive, it could be a nucleophilic attack.

The Fukui functions of the molecules (a–d) are calculated with the help of Multiwfn based on the energies calculated by the B3LYP/6-31 G (d, p) method and listed in Table 4 and Table 5.

According to Table 4, the descriptor values for heptanal (a) and linoleic acid (b) indicate that the sites of nucleophilic attack are O1, C8, H17, H18 and O1, O2, C7, C9, C16, C18, H22, H25, H26, H29, H30, H33, H34, H46, H52, respectively, while the electrophilic attack is on other atoms of the molecules. According to Table 5, the descriptor values for camphene (c) and α-humulene (d) indicate that the sites of nucleophilic attack are C2, C5, H11, H13, H15, H18–H21, H24, H26 and C3–C9, C11, C14, H17–H29, H35, respectively, while the electrophilic attack is on other atoms of the molecules. The high value of an atom’s Fukui function indicates high molecular reactivity [46]. 

In this case, the most reactive atoms of heptanal (a), linoleic acid (b), camphene (c), and α-humulene (d) are O1, C12, C6, and C13 for an electrophilic attack; are C8, C18, C6, and C9 for a nucleophilic attack; and are O1, O2, C6 for the radical attack, respectively.

## 4. Conclusions

In this study, *Aethionema sancakense* plant, which was determined as a new species and had its essential oil and fatty acid compositions characterized by GC/GC-MS technique, is reported. Major essential oil constituents of the aerial part structures of this plant were determined as linoleic acid (23.1%), α-humulene (19.8%), camphene (13.9%), and heptanal (9.7%). Theoretical work was then carried out to better understand the structures of these four important molecules. For this purpose, the optimized molecular geometries of the molecules were determined first and then the molecular electrostatic potential (MEP), frontier moleculer orbitals (FMOs), and Fukui analysis were performed to determine the electronic properties and sites of nucleophilic and electrophilic attacks. Finally, the RDG-NCI analysis of these molecules was performed to determine the non-covalent interactions present within the molecules. According to the results, we can say that molecule a is the most reactive molecule and molecule c has a stronger steric effect than other molecules.

The theoretical studies obtained, along with the works [47,48,49,50,51,52], may influence the understanding of some interactions and properties of natural compounds in the future.

## Figures and Tables

**Table 1:** Constituents of the essential oil from *Aethionema sancakense*.

No.	RII	Name of Compounds	Area
1	901	3-Hexanone	0.1
2	904	2-Heptanone	0.8
3	914	**Heptanal**	**9.7**
4	952	**Camphene**	**13.9**
5	959	Benzaldehyde	0.7
6	1003	α-Phellandrene	1.3
7	1024	Geraniol	3.4
8	1042	Benzene acetaldehyde	3.5
9	1090	p-Cymene	4.7
10	1358	β-Cubebene	1.8
11	1385	β-Bourbonene	1.4
12	1392	Caryophyllene	2.1
13	1450	**α-Humulene**	**19.8**
14	1485	β-Selinene	4.3
15	1495	Viridiflorene	1.2
16	1496	Caryophyllene oxide	3.0
17	1594	Benzyl benzoate	0.5
18	1598	Estragole	0.3
19	1629	2-Pentadecanone	1.7
20	1643	n- Hexadecanoic acid	2.7
21	1900	**Linoleic acid**	**23.1**
**Total**			**100.0**
		Monoterpenes	38.1
		Sesquiterpenes	33.6
		Others	28.3

**Table 2:** Fatty acid composition (%) of *Aethionema sancakense*.

Studied Sample	Palmitic Acid C 16:0	Pentadecanoic Acid C 15:0	Stearic Acid C 18:00	Oleic Acid C 18:1	Linoleic Acid C 18:2	Linolenic Acid C 18:3	Eikosadienoic Acid C 20:2	TOTAL
Aethionema sancakense	14.70	0.18	2.76	1.51	55.38	9.26	1.93	75.72

**Table 3:** Some electronic properties of studied molecules (a–d) calculated by the DFT/B3LYP method in the gas phase and water *.

Parameters	Values (eV)							
	a	a *	b	b *	c	c *	d	d *
E_HOMO_	−6.8448	−6.9441	−6.3634	−6.4494	−6.3569	−6.4502	−5.8143	−5.8627
E_LUMO_	−0.5116	−0.5423	0.3271	0.2522	0.7423	0.6574	0.6055	0.5619
Egap	6.3332	6.4018	6.6905	6.7016	7.0992	7.1076	6.4197	6.4246
IP	6.8448	6.9441	6.3634	6.4494	6.3569	6.4502	5.8143	5.8627
EA	0.5116	0.5423	−0.3271	−0.2522	−0.7423	−0.6574	−0.6055	−0.5619
χ	3.6782	3.7432	3.0182	3.0986	2.8073	2.8964	2.6044	2.6504
μ	−3.6782	−3.7432	−3.0182	−3.0986	−2.8073	−2.8964	−2.6044	−2.6504
ζ	0.3158	0.3124	0.2989	0.2984	0.2817	0.2814	0.3115	0.3113
η	3.1666	3.2009	3.3452	3.3508	3.5496	3.5538	3.2099	3.2123
ω	2.1362	2.1887	1.3615	1.4327	1.1101	1.1803	1.0566	1.0934
∆N_max_	1.1616	1.1694	0.9022	0.9247	0.7909	0.8150	0.8114	0.8251
N	0.4681	0.4569	0.7345	0.6980	0.9008	0.8473	0.9465	0.9146

E_HOMO_: energy of HOMO; E_LUMO_: energy of LUMO; Egap: energy gap; IP: ionization potential; EA: electron affinity; χ: electronegativity; μ: chemical potential; ζ: chemical softness; η: chemical hardness; ω: global electrophilicity index; ∆N_max_: maximum charge transfer index; N: nucleophilicity index.

**Table 4:** The condensed Fukui functions for heptanal (a) and linoleic acid (b).

(a)					(b)				
Atoms	$f_{r}^{-}$	$f_{r}^{+}$	$f_{r}^{0}$	Δf(r)	Atoms	$f_{r}^{-}$	$f_{r}^{+}$	$f_{r}^{0}$	Δf(r)
1(O)	0.2518	0.2627	0.2573	0.0109	1(O)	0.0327	0.0534	0.0431	0.0207
2(C)	0.0301	0.0103	0.0202	−0.0199	2(O)	0.0726	0.0955	0.0841	0.0229
3(C)	0.0338	0.0064	0.0201	−0.0274	3(C)	0.0057	0.0039	0.0048	−0.0018
4(C)	0.0221	0.0073	0.0147	−0.0148	4(C)	0.007	0.0056	0.0063	−0.0014
5(C)	0.0284	0.0051	0.0167	−0.0233	5(C)	0.0056	0.005	0.0053	−6.00 × 10^−4^
6(C)	0.0571	0.0475	0.0523	−0.0096	6(C)	0.0119	0.007	0.0094	−0.0049
7(C)	0.0329	0.0069	0.0199	−0.026	7(C)	0.0032	0.0034	0.0033	3.00 × 10^−4^
8(C)	0.1137	0.2981	0.2059	0.1845	8(C)	0.0151	0.011	0.0131	−0.0041
9(H)	0.0206	0.0133	0.0169	−0.0074	9(C)	0.0115	0.0198	0.0156	0.0083
10(H)	0.0206	0.0133	0.0169	−0.0074	10(C)	0.011	0.0065	0.0087	−0.0045
11(H)	0.0185	0.0075	0.013	−0.011	11(C)	0.006	0.0046	0.0053	−0.0014
12(H)	0.0185	0.0075	0.013	−0.011	12(C)	0.0936	0.0664	0.08	−0.0271
13(H)	0.0183	0.0134	0.0159	−0.0049	13(C)	0.0146	0.0109	0.0127	−0.0037
14(H)	0.0183	0.0134	0.0158	−0.0049	14(C)	0.0047	0.0025	0.0036	−0.0022
15(H)	0.0176	0.0059	0.0118	−0.0118	15(C)	0.0812	0.0544	0.0678	−0.0268
16(H)	0.0176	0.0059	0.0118	−0.0118	16(C)	0.016	0.018	0.017	0.002
17(H)	0.0445	0.069	0.0567	0.0246	17(C)	0.0936	0.0713	0.0824	−0.0222
18(H)	0.0445	0.069	0.0567	0.0246	18(C)	0.024	0.1014	0.0627	0.0774
19(H)	0.0401	0.0126	0.0263	−0.0275	19(C)	0.0819	0.0561	0.069	−0.0259
20(H)	0.0175	0.0058	0.0116	−0.0117	20(C)	0.0061	0.0032	0.0047	−0.003
21(H)	0.0175	0.0058	0.0116	−0.0117	21(H)	0.0065	0.0057	0.0061	−7.00 × 10^−4^
22(H)	0.116	0.1135	0.1148	−0.0025	22(H)	0.0027	0.0032	0.0029	5.00 × 10^−4^
					23(H)	0.0094	0.0079	0.0087	−0.0015
					24(H)	0.005	0.0045	0.0048	−5.00 × 10^−4^
					25(H)	0.0044	0.0055	0.005	0.0011
					26(H)	0.0068	0.0072	0.007	5.00 × 10^−4^
					27(H)	0.0116	0.0089	0.0103	−0.0027
					28(H)	0.0041	0.004	0.0041	−1.00 × 10^−4^
					29(H)	0.0029	0.0055	0.0042	0.0026
					30(H)	0.0049	0.0068	0.0058	0.0019
					31(H)	0.0103	0.0076	0.009	−0.0027
					32(H)	0.0286	0.0211	0.0248	−0.0075
					33(H)	0.0117	0.0303	0.021	0.0185
					34(H)	0.0105	0.0291	0.0198	0.0186
					35(H)	0.0032	0.0022	0.0027	−0.001
					36(H)	0.012	0.0097	0.0109	−0.0023
					37(H)	0.0094	0.0082	0.0088	−0.0011
					38(H)	0.0028	0.0012	0.002	−0.0016
					39(H)	0.0359	0.0292	0.0326	−0.0067
					40(H)	0.0087	0.0063	0.0075	−0.0024
					41(H)	0.0294	0.0225	0.026	−0.0069
					42(H)	0.0071	0.0062	0.0067	−9.00 × 10^−4^
					43(H)	0.0013	−4.00 × 10^−4^	5.00 × 10^−4^	−0.0017
					44(H)	0.0311	0.0263	0.0287	−0.0048
					45(H)	0.0135	0.0112	0.0124	−0.0024
					46(H)	0.0258	0.0348	0.0303	0.0089
					47(H)	0.0362	0.031	0.0336	−0.0052
					48(H)	0.032	0.0267	0.0293	−0.0054
					49(H)	0.0073	0.0063	0.0068	−0.001
					50(H)	0.0096	0.0058	0.0077	−0.0038
					51(H)	0.0018	−0.0014	2.00 × 10^−4^	−0.0032
					52(H)	0.0154	0.0268	0.0211	0.0114

**Table 5:** The condensed Fukui functions for camphene (c) and α-humulene (d).

(c)					(d)				
Atoms	$f_{r}^{-}$	$f_{r}^{+}$	$f_{r}^{0}$	Δf(r)	Atoms	$f_{r}^{-}$	$f_{r}^{+}$	$f_{r}^{0}$	Δf(r)
1(C)	0.0148	0.0089	0.0119	−0.0059	1(C)	0.0104	0.0101	0.0103	−2.00 × 10^−4^
2(C)	0.0139	0.0139	0.0139	1.00 × 10^−4^	2(C)	0.0189	0.0181	0.0185	−7.00 × 10^−4^
3(C)	0.0294	0.0232	0.0263	−0.0062	3(C)	0.0401	0.0738	0.057	0.0337
4(C)	0.0196	0.0175	0.0186	−0.0021	4(C)	0.0143	0.0164	0.0153	0.0021
5(C)	0.1282	0.1429	0.1356	0.0147	5(C)	0.0063	0.0094	0.0078	0.0031
6(C)	0.216	0.1987	0.2074	−0.0173	6(C)	0.0603	0.062	0.0611	0.0018
7(C)	0.0251	0.0211	0.0231	−0.004	7(C)	0.0542	0.057	0.0556	0.0028
8(C)	0.0334	0.0286	0.031	−0.0048	8(C)	0.0131	0.015	0.0141	0.002
9(C)	0.0169	0.0132	0.0151	−0.0037	9(C)	0.0395	0.0752	0.0574	0.0357
10(C)	0.0248	0.0188	0.0218	−0.0061	10(C)	0.0168	0.0164	0.0166	−4.00 × 10^−4^
11(H)	0.0274	0.0285	0.0279	0.0011	11(C)	0.0171	0.0203	0.0187	0.0031
12(H)	0.0172	0.0152	0.0162	−0.002	12(C)	0.0857	0.0352	0.0604	−0.0505
13(H)	0.0368	0.0381	0.0375	0.0014	13(C)	0.089	0.0294	0.0592	−0.0596
14(H)	0.0187	0.0167	0.0177	−0.0021	14(C)	0.0133	0.0168	0.015	0.0034
15(H)	0.0348	0.0349	0.0348	2.00 × 10^−4^	15(C)	0.022	0.0154	0.0187	−0.0067
16(H)	0.0152	0.0143	0.0147	−9.00 × 10^−4^	16(H)	0.0347	0.027	0.0308	−0.0077
17(H)	0.0162	0.0145	0.0153	−0.0017	17(H)	0.0139	0.0144	0.0141	4.00 × 10^−4^
18(H)	0.0644	0.0731	0.0687	0.0087	18(H)	0.0141	0.0288	0.0215	0.0147
19(H)	0.0617	0.0709	0.0663	0.0092	19(H)	0.0179	0.0292	0.0236	0.0113
20(H)	0.0273	0.0329	0.0301	0.0056	20(H)	0.0108	0.0141	0.0125	0.0033
21(H)	0.041	0.0574	0.0492	0.0164	21(H)	0.0171	0.018	0.0175	8.00 × 10^−4^
22(H)	0.0184	0.0175	0.018	−9.00 × 10^−4^	22(H)	0.0169	0.0227	0.0198	0.0058
23(H)	0.0183	0.0168	0.0175	−0.0015	23(H)	0.0147	0.015	0.0148	3.00 × 10^−4^
24(H)	0.0305	0.0305	0.0305	0	24(H)	0.0026	0.0092	0.0059	0.0066
25(H)	0.018	0.0173	0.0177	−7.00 × 10^−4^	25(H)	0.0225	0.0269	0.0247	0.0044
26(H)	0.0322	0.0346	0.0334	0.0024	26(H)	0.0263	0.0338	0.03	0.0075
					27(H)	0.0158	0.0164	0.0161	7.00 × 10^−4^
					28(H)	0.0209	0.0331	0.027	0.0122
					29(H)	0.0331	0.0355	0.0343	0.0024
					30(H)	0.017	0.0143	0.0156	−0.0027
					31(H)	0.0326	0.031	0.0318	−0.0017
					32(H)	0.0273	0.0213	0.0243	−0.006
					33(H)	0.0229	0.015	0.019	−0.0079
					34(H)	0.0147	0.014	0.0143	−7.00 × 10^−4^
					35(H)	0.0131	0.0183	0.0157	0.0052
					36(H)	0.0273	0.0327	0.03	0.0054
					37(H)	0.0312	0.0157	0.0235	−0.0155
					38(H)	0.0332	0.0274	0.0303	−0.0059
					39(H)	0.0185	0.0156	0.0171	−0.0028

## Data Availability

Not applicable.
